# Facile Processing of Transparent Wood Nanocomposites with Structural Color from Plasmonic Nanoparticles

**DOI:** 10.1021/acs.chemmater.1c00806

**Published:** 2021-05-04

**Authors:** Martin Höglund, Jonas Garemark, Mathias Nero, Tom Willhammar, Sergei Popov, Lars A. Berglund

**Affiliations:** † Department of Fibre and Polymer Technology, Wallenberg Wood Science Center, KTH Royal Institute of Technology, SE-100 44 Stockholm, Sweden; ‡ Department of Materials and Environmental Chemistry, Stockholm University, SE-106 91 Stockholm, Sweden; § Department of Applied Physics, KTH Royal Institute of Technology, 114 19 Stockholm, Sweden

## Abstract

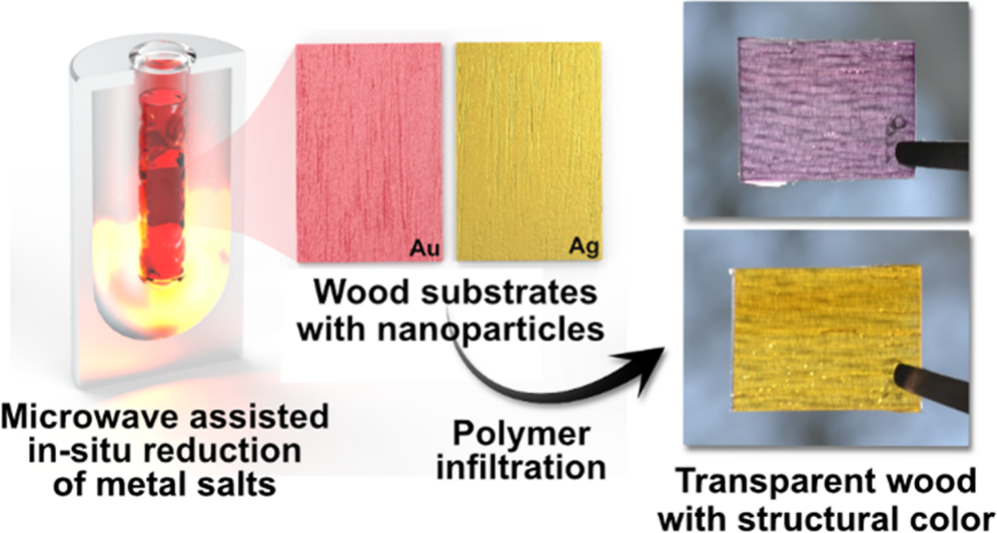

Wood is an eco-friendly and abundant substrate and a candidate for functionalization by large-scale nanotechnologies. Infiltration of nanoparticles into wood, however, is hampered by the hierarchically structured and interconnected fibers in wood. In this work, delignified wood is impregnated with gold and silver salts, which are reduced in situ to plasmonic nanoparticles via microwave-assisted synthesis. Transparent biocomposites are produced from nanoparticle-containing wood in the form of load-bearing materials with structural color. The coloration stems from nanoparticle surface plasmons, which require low size dispersity and particle separation. Delignified wood functions as a green reducing agent and a reinforcing scaffold to which the nanoparticles attach, predesigning their distribution on the surface of fibrous “tubes”. The nanoscale structure is investigated using scanning transmission electron microscopy (STEM), energy-dispersive spectroscopy (EDS), and Raman microscopy to determine particle size, particle distribution, and structure–property relationships. Optical properties, including response to polarized light, are of particular interest.

## Introduction

Plasmonic nanoparticles (PNPs) are of great interest as constituents in complex nanomaterials and can provide functions exemplified by photonic materials for e.g., optoelectronic devices,^1^ solar energy management,^2^ or provision of structural color.^3^ Although sophisticated films and coatings with highly ordered PNP structures have been produced,^4^ they tend to be mechanically delicate, and the challenge of large-scale processing needs to be addressed for plasmonic nanomaterials in general.^5^ Recently, wood was suggested as a substrate in large-scale nanotechnologies,^6^ as exemplified by structural EMI-shielding,^7^ since it is a low-cost, eco-friendly structural material with a sophisticated hierarchical structure. Combinations of chemical modification and embedding of inorganic nanoparticles can add new functionalities to wood, in an attractive combination with its structural properties. In the present study, a processing method for load-bearing wood biocomposites with PNPs is reported, and the resulting nanocomposites are investigated for their optical properties and structural color effects.

Transparent wood (TW) is a new form of wood biocomposites, which has garnered interest as a material combining optical transmittance with mechanical performance and lightweight.^8,9^ This top-down approach utilizes the existing, oriented cellulose structures in wood, as a simpler alternative to bottom-up freeze-dried cellulose structures,^10^ where processing-related energy demands will be much higher. TW is created by chemical removal of lignin chromophores, followed by impregnation and polymerization of a polymer with a matching refractive index to the wood structure. It was first investigated in anatomical studies of wood,^11^ but the recent focus is on multifunctional biocomposites, including stretchable/conductive,^12^ magnetic,^13^ heat shielding,^14^ and thermal energy storing TW,^15^ smart windows,^16^ and in solar energy applications.^17,18^ Further studies have shown their potential as a photonic nanomaterial, by incorporation of luminescent dyes,^19−21^ quantum dots^22^ and photochromic^23^ or photo/thermochromic compounds.^24^ Interesting optical effects have been observed such as anisotropic light scattering,^25,26^ wave guiding,^20^ polarization effects,^27^ and wood cells functioning as optical gain cavities for lasing.^21^ Chemical stability is a concern for PNPs,^28^ but the present thiol-ene polymer improves chemical stability by antioxidative properties,^29^ and reduces particle agglomeration problems.^30^

A class of interesting optical functions, not investigated for TW, is structural coloration. Structural colors are renowned for their vibrancy, iridescence, and photostability. Brilliant colors found in butterfly wings, beetle shells, fish scales, and bird feathers all stem from their micro to nanoscale structures.^31^ Structural colors are related to micro- and nanoscale structures, which interact with light, contrary to dyes which rely on absorption by their specific molecular spectra. In plasmonic materials, conduction band electrons oscillate coherently in response to external illumination, generating a responding electric field at the surface. The modes of oscillation are known as surface plasmons. Incident electromagnetic waves which resonate with the surface plasmons are strongly absorbed or scattered, observed as absorption bands originating from surface plasmon resonance (SPR).^32^ SPRs depend on the dielectric character of the material, as well as the spatial confinement in terms of size and geometry of the nanoparticles,^32−34^ and is therefore considered a subset of structural coloring.^35^ Gold and silver nanoparticles have SPRs within the range of visible light and produce brilliant structural colors, and exhibit molar extinction coefficients, which are several times higher than common organic pigments.^32,36^ In addition, common pigments suffer from photodegradation, which noble metals avoid due to their excellent stability and different origin of the coloring response.^32,35^ Plasmonic color does, however, require careful control of particle size dispersion, and geometry, to produce narrow SPR bands.

PNPs have been used empirically throughout history to produce brilliant colors in ceramic glazes,^37^ stained church windows,^38^ and in early photography.^39^ Today, interest in PNPs is due to their photostability and strong response to the exciting light, which could decrease colorant load and remove the need for re-coloration. PNPs have therefore been proposed for textile coloration,^40−43^ and are of interest for photonic biocomposites. Furthermore, PNPs have shown potential as active species in optical filters and polarizers for information technology, in nonlinear optics, for surface-enhanced fluorescence, and for improved photovoltaic devices for solar cell applications.^44,45^

Wood has been functionalized with PNPs for water treatment applications, either as filters^46^ or for solar steam generation,^47,48^ but never for coloration purposes where low particle size variation and good dispersion are required.^32^ To the best of our knowledge, structural coloration of wood has only been proposed as surface coatings of photonic crystals^49,50^ or alternating layers of mica and titania.^51^ Such structures display angle-dependent colors, unlike PNP coloration.

Studies on water treatment applications focused primarily on bactericidal^46^ and steam generation functions, with limited analysis of nanostructural features.^47,48^ Harmful reducing agents were often added to convert metal salts to nanoparticles. An interesting, more eco-friendly approach is suggested, where wood-derived compounds are utilized as green reducing agents. Cellulose hydrogels,^52^ cellulose derivatives,^53^ lignocellulosic fibers,^54^ cellulose nanocrystals,^55^ wood powders,^56^ and bleached pulp^40^ have been used to reduce metal salts. To our knowledge, only palladium particles have been precipitated in wood without reducing agents,^57^ where wood lignin served as a reducing agent. Lignin is, however, commonly removed during TW preparation,^8,9^ since it contains wood chromophores.^58^ In the present study, delignified wood is by itself reducing silver and gold salts to PNPs.

Hydrothermal synthesis of metal nanoparticles often requires reaction times of several hours when heated by standard means.^40,47,52,55,57^ In microwave-assisted synthesis, inverted heat gradients are efficiently and rapidly heating bulk solutions.^59,60^ Reaction times are drastically reduced (5–10 min for gold and silver nanoparticles) and dispersions of smaller particle size are obtained due to improved nucleation.^60^ The porous structure of wood is believed to improve electromagnetic absorption and conversion to thermal energy,^61^ and biomass-derived materials have been used for electromagnetic shielding.^62,63^ In previous investigations, iron oxide particles were formed inside wood.^64^ A variety of metal nanoparticles were precipitated on bacterial cellulose fibrils, and combined into multifunctional laminate structures;^65^ a related approach was demonstrated for wood-based aerogels.^66^

The hierarchical and anisotropic wood structure is a specific characteristic of TW biocomposites, resulting in mechanical^67^ and optical anisotropy.^25−27^ Functional anisotropy can also be designed by selective deposition of nanoparticles on nanofibers,^68^ and/or on cell walls in a wood substrate. The particle distribution becomes predesigned by the wood structure and, thus, mimics its anisotropy. In a previous study,^69^ magnetic nanoparticles were found to decorate the inside of wood fibers, but anisotropic predesign of TW using nanoparticles has not been investigated in detail. In the present study, the distribution of PNPs is scrutinized and related to enhanced anisotropic optical effects.

TW with structural color requires well-dispersed PNPs with nanostructurally controlled sizes, since the SPR scattering bands are sensitive to particle aggregation.^32^ The main objectives are therefore to tailor and control the processes during both in situ synthesis of PNPs and TW fabrication. In addition, relationships between composition/structure and physical properties are investigated. The mechanisms of particle formation and separation are explored, in particular the role of delignified wood as a green reducing agent and scaffold for controlled PNP nanoparticle distribution. After TW fabrication, the resulting brilliantly colored TW nanocomposites combine load-bearing and also functional properties, in the form of anisotropic optical properties and polarization effects.

## Experimental Section

### Materials

Rotary cut balsa veneers with a density of 90–110 kg m^–3^ was supplied by Material AB (Sweden). Sodium acetate, silver nitrate (AgNO3), chloroauric acid (HAuCl4), pentaerythritol tetrakis(3-mercaptoproponiate) (PETMP), 1,3,5-trially-1,3,5-trione-2,4,6(1*H*,3*H*,5*H*)-triazine (TATATO), and 1-hydroxycyclohexyl phenyl ketone were all supplied by Merck. Acetone and ethanol absolute were supplied from VWR. Acetic acid (Honey-well) and sodium chlorite (Alfa Aesar) were supplied by other vendors.

### Wood Delignification

Balsa veneers were cut with a razor blade to 1.5 × 1.2 × 0.1 cm^3^ (for optical characterization) or 5.0 × 0.5 × 0.1 cm^3^ (for tensile testing) pieces. The pieces were delignified at 80 °C in acetate buffer containing sodium chlorite (1 wt %), according to a previously published method.^8^ Delignification continued until the pieces turned completely white. The delignified substrates were subsequently washed in deionized water.

### Microwave-Assisted In Situ Synthesis of PNPs

Delignified wood substrates were infiltrated under reduced pressure with aqueous solutions (4 mL) of either silver nitrate (0.5 mM) or chloroauric acid (0.1 mM) for 3 h. Infiltrated substrates were kept in metal salt solutions during in situ synthesis, which was performed with a temperature-controlled single-mode (2.45 GHz) microwave oven (Biotage Initiator). Samples were heated to and kept at 120 °C during synthesis, followed by cooling down to 50 °C. The reaction time was 5 min for chloroauric solutions and 10 min for silver nitrate solutions.

### Transparent Wood Preparation

TW preparation followed a fabrication method reported elsewhere.^70^ Substrates were sequentially solvent exchanged from deionized water to ethanol absolute and to acetone. A stoichiometric mixture, according to functional groups, of TATATO and PETMP containing 1-hydroxycyclohexyl phenyl ketone (0.5 wt %) was infiltrated into the substrates for at least 4 h inside a fume hood. Impregnation was mediated by acetone, which was then evaporated. UV illumination for 4 min using four 9W 365 nm UV-lamps, cured the thiol-ene thermoset, resulting in TW biocomposites.

### Characterization

Wood content in finished TW composites was calculated from the average density of delignified substrates and the dimensions and weight of TW samples.

A Universal Testing Machine (Instron 5566) equipped with a video extensometer was used to perform tensile tests with a 10 kN load cell, 5 mm min^–1^ piston speed, and a grip distance of 2.5 mm. Samples (5.0 × 0.5 × 0.1 cm^3^) were conditioned at 22 °C and 50% relative humidity overnight prior to testing. The presented data are averages of five samples per specific composite.

Total transmittance (ballistic and diffuse light) was measured according to ASTM D1003-13.^71^ TW samples were placed at the opening port of an integrating sphere and exposed to spectrally broadband light (quartz tungsten halogen light, model 66181, Oriel Instruments). The same set-up was used to measure polarized transmittance by placing a polarizing filter between the sample and the integrating sphere. The samples were measured with the wood fibers oriented parallel and perpendicular to the polarization direction of the filter. The ratio of polarization split is the quotient of the two polarized measurements.

Scanning transmission electron microscopy (STEM) images of the TW materials were acquired using a double aberration-corrected Thermo Fisher Themis-Z transmission electron microscope. The microscope was operated at an accelerating voltage of 300 kV. Annular dark-field (ADF) STEM images were acquired using a beam current of 40 pA, a beam convergence angle of 21 mrad, an inner collection angle of 39 mrad, and a dwell time of 20 μs.

To study the interior of the TW material, ultra-thin transverse sectioning was carried out using a Leica Ultracut UCT with a 45° diamond knife from Diatome. A cutting speed of 1 mm s^–1^ was used and a clearance angle of 6°. The sections have estimated thicknesses of ca. 100 nm. After cutting, the sections were transferred to carbon coated copper grids (EMS CF400-CU-UL) for STEM analysis. No staining or other contrast enhancing chemicals were used.

Scanning electron microscopy (SEM) imaging and energy-dispersive X-ray spectroscopy (EDS) were conducted with a field emission SEM (FE-SEM) (Hitachi S-4800, Japan) with an attached EDX probe (X-MaxN, Oxford Instruments). Freeze fractured wood substrates with a 1 nm platinum/palladium coating were used for cell wall imaging. Micrographs were captured at an accelerating voltage of 15 kV, an amperage of 2 μAm, and a working distance of 5 cm. EDS mapping was performed on uncoated substrate fractures, using 6 kV, 20 μA, and a working distance of 15 cm.

Raman spectra of the cell wall surfaces of wood substrates were measured with a confocal Raman microscope (Jobin Yvon HR800 UV, Horiba) using a 514 nm laser (Stellar-Pro, Modu-laser). Spectra were accumulatively acquired 16 times, baselined and normalized to CH_2_ vibrations at 1333 cm^–1^.

X-ray diffraction (XRD) measurements were carried out on wood substrates, compressed with 15 MPa for 2 min, with a powder X-ray diffractometer (ARL X’TRA, Thermo Fisher Scientific). Scans over 2θ of 5–50°, with a 0.04 step size, were performed with Cu Kα radiation at 40 mA and 45 kV. The peak height method was used to calculate the crystallinity index (CI) as the quotient of the crystalline [200] peak and the total peak intensity (I200):^72^
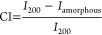
1Fourier transformed infrared spectroscopy (FTIR) was conducted on wood substrates using a Spectrum 100 FTIR (PerkinElmer) with a MKII Golden Gate ATR system (Specac Ltd.) Spectra were accumulatively acquired 16 times.

## Results and Discussion

### Materials Processing and Mechanical Properties

**Figure 1:**
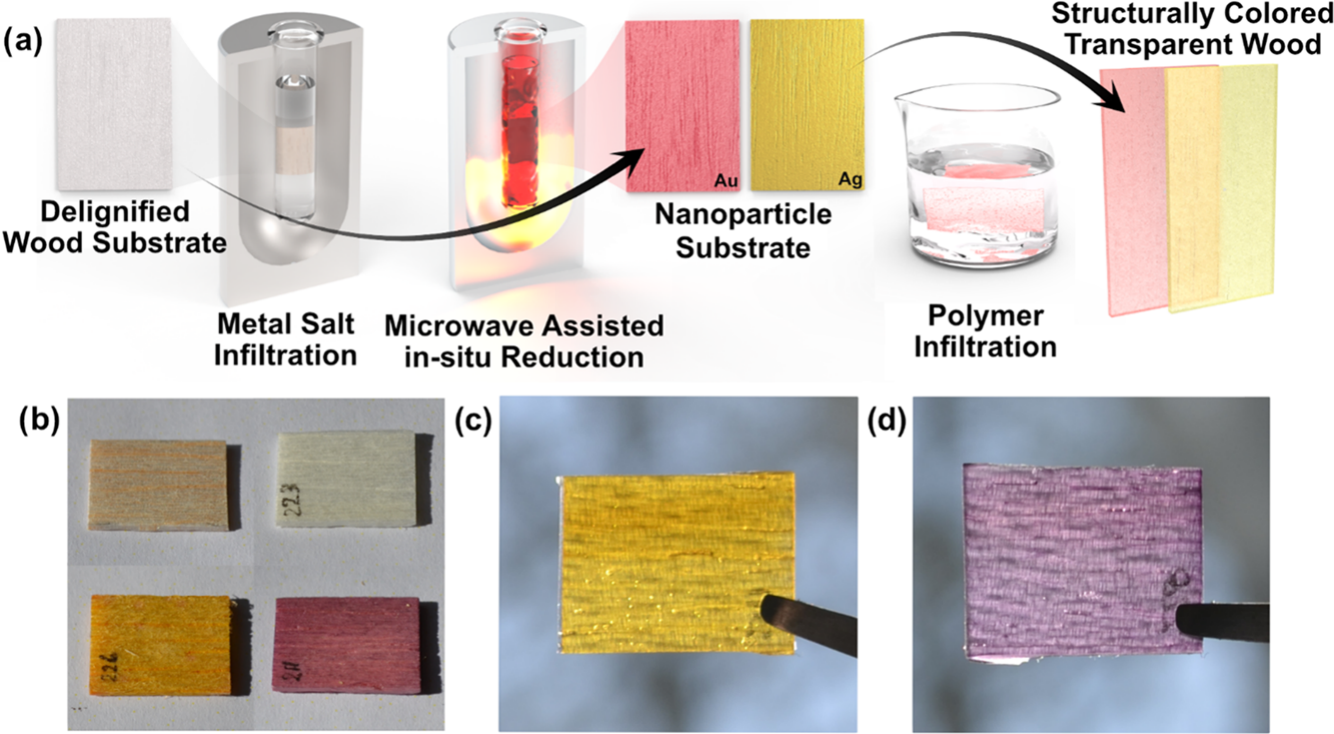
(a) Schematic sketch of structurally colored TW processing: delignified wood is infiltrated with metal salts (silver or gold) which are in situ reduced to plasmonic nanoparticles by microwave-assisted synthesis. Nanoparticle-containing substrates are then infiltrated with monomers and cured to TW composites with structural color. Photographs of (b) balsa wood, delignified substrate, silver substrate, gold substrate, (c) Ag-TW, and (d) Au-TW.

Structurally colored transparent wood was prepared by in situ synthesis of plasmonic nanoparticles (PNPs) in delignified wood substrates, followed by impregnation and curing of a thiol-ene polymer matrix. Delignification of balsa veneers was first carried out to remove chromophores,^8^ producing bright white wood substrates (Figure 1b). The substrates were subsequently washed in deionized water and infiltrated with aqueous solutions of 0.1 mM chloroauric acid or 0.5 mM silver nitrate. Higher metal salt concentrations were tested but resulted in inferior colors for gold substrates (Figure S1), likely due to the formation and agglomeration of larger particles. Silver templates were seemingly unaffected within the range of concentrations. Silver NPs are possibly more stable at the reaction temperature than gold due to higher binding energy to carboxyl groups,^73,74^ which are formed during synthesis. Substrates remained white until subjected to microwave-assisted synthesis of nanoparticles. PNP formation is apparent from the brilliant colors produced by the substrates; bright yellow silver and pinkish-purple gold (Figure 1c,d). Lightly colored and stable nanoparticle suspensions were also produced in the process. No additional reducing agent was needed, as wood compounds function as green reducing agents. Any degradation of the substrate was superficial and structural integrity was retained. In situ synthesis of PNPs is, thus, a potential method for preparing wood with structural color.

To provide optical transmittance and improve mechanical stability, a polymer matrix with a matching refractive index replaced voids in the porous substrate.^8,70^ A UV-curable thiol-ene thermoset, previously used for TW preparation,^70^ was used. Impregnation of thiol-ene precursors was mediated by solvent-assisted impregnation. Substrates were sequentially exchanged from deionized water to ethanol and acetone prior to precursor impregnation. Acetone was then evaporated prior to curing. PNPs were well attached to the substrate, and neither loss in substrate color, nor color acquisition of the polymer, was observed. Curing of infiltrated precursors resulted in brilliantly colored transparent wood (TW) biocomposites, i.e., bright yellow Ag-TW (silver) and purple Au-TW (gold), as presented in Figure 1. The final composites have a wood content of 6.8 vol % and minuscule amounts of nanoparticles, 0.02 vol % silver and 0.01 vol % gold. The low nanoparticle content demonstrates the strong coloration effect from PNPs.

TW composites exhibit favorable anisotropic mechanical^67^ and optical properties,^25−27^ which are linked to the wood structure. Hardwoods (present class of wood species) consist primarily of fibers, vessels, and ray cells, all tubular in structure. Fibers and vessels are oriented parallel to the tree stem (longitudinal direction), and in Figure 1c,d this is the horizontal direction of the TW composites. Fibers dominate the wood structure and provide mechanical support. Vessels are fewer but larger in size and facilitate transport of liquids and nutrients. Rays are oriented perpendicular to the longitudinal direction and results in liquid transport (outward from the center of the tree) and storage of nutrients. Rays appear as dark streaks in Figure 1c,d. Balsa also contains high fractions of longitudinal parenchyma cells, which mainly store nutrients. Hardwoods thus show an anisotropic structure with high strength in the longitudinal direction.^67^ In TW biocomposites, the wood substrate thus provides mechanical reinforcement.^8,9^ It is also a scaffold controlling nanoparticle distribution in the present study and serves as a green reducing agent during PNP formation.

Tensile properties are summarized in Table 1 and stress–strain curves are in Figure S2. Young’s modulus (4.11 GPa), tensile strength (50.7 MPa), and strain to failure (1.2%) of reference TW were not significantly impacted by incorporation of PNPs (3.80 GPa Young’s modulus, 45.7 MPa tensile strength, and 1.3% strain to failure for Ag-TW). The slight lowering of mechanical properties is probably from local density variations in wood, rather than a real effect from structural changes. In situ synthesis of NPs is deemed to have a low to negligible impact on the mechanical properties of TW.

**Table 1:** Tensile Properties^a^

	Young’s modulus [GPa]	tensile strength [MPa]	strain to failure [%]
Ag-TW	3.80 ± 0.62	45.7 ± 2.5	1.3 ± 0.2
TW	4.11 ± 0.50	50.7 ± 2.5	1.2 ± 0.1

a Averaged values from five samples per composite.

### Optical Properties

**Figure 2:**
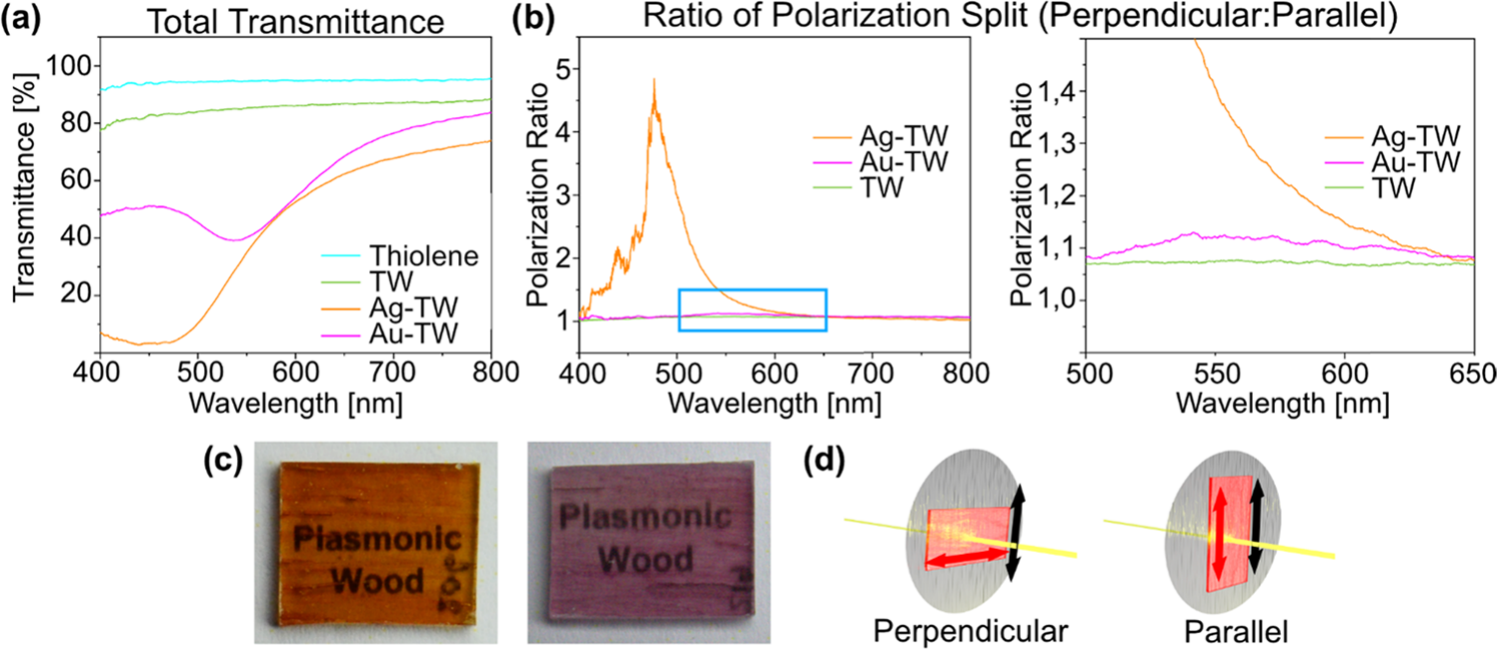
Optical properties of Ag-TW and Au-TW: (a) total transmittance and (b) ratio of polarization split. (c) Photographs of Ag-TW and Au-TW with visible text underneath. (d) Specimen set-up for polarized transmittance measurements of perpendicular and parallel orientation.

Optical properties of structurally colored TW are interesting in several aspects. Light absorption and scattering, as well as optical effects associated with material anisotropy are defined by both the composite components (wood substrate and polymer) and the added metal nanoparticles. Optical transmittance (Figure 2a) was characterized using thin TW samples (1.2 mm) and an integrating sphere set-up, making scattering losses negligible.^26^ A reference TW (green line in Figure 2a) demonstrated lower transmittance than neat thiol-ene (blue line in Figure 2a), as expected from wood substrate scattering effects.

Addition of metal nanoparticles into the host matrix significantly changes the optical transmission. Metal nanoparticles themselves exhibit band absorption caused by the excitation of surface plasmons with surface plasmon resonances (SPR) in the visible range. The specific SPR wavelength is related to the size, geometry, and material of the nanoparticle.^34,75^ The broadening of the SPR extinction band is defined by the size dispersion of nanoparticles. Transmittance spectra of structurally colored TW are presented in Figure 2a. The minimum transmission for both materials corresponds to the SPR of the respective material, 438 nm (silver) and 538 nm (gold). A stronger absorption of Ag-TW is related to a higher nanoparticle content in the TW substrate and higher electrical conductivity of silver.^34^

Interestingly, the polarization of transmitted light is also affected by the presence of nanoparticles. Pure TW (without nanoparticles) exhibits variation in light transmission for different polarizations,^27^ which was also observed for the reference TW (green line in Figure S3). The light which is polarized perpendicular to the direction of fibers demonstrated a slightly higher transmission, as compared with light polarized along the wood fibers. This effect can be attributed to the spatial anisotropy of the wood structure. It resembles a diffraction grating with a uniform structure parallel to the fiber direction, but poor periodicity perpendicular to the fiber orientation. Thus, after light propagation through the material, the light polarization split replicates the polarization split in conventional diffraction gratings, although with a less noticeable effect.^76^

**Figure 3:**
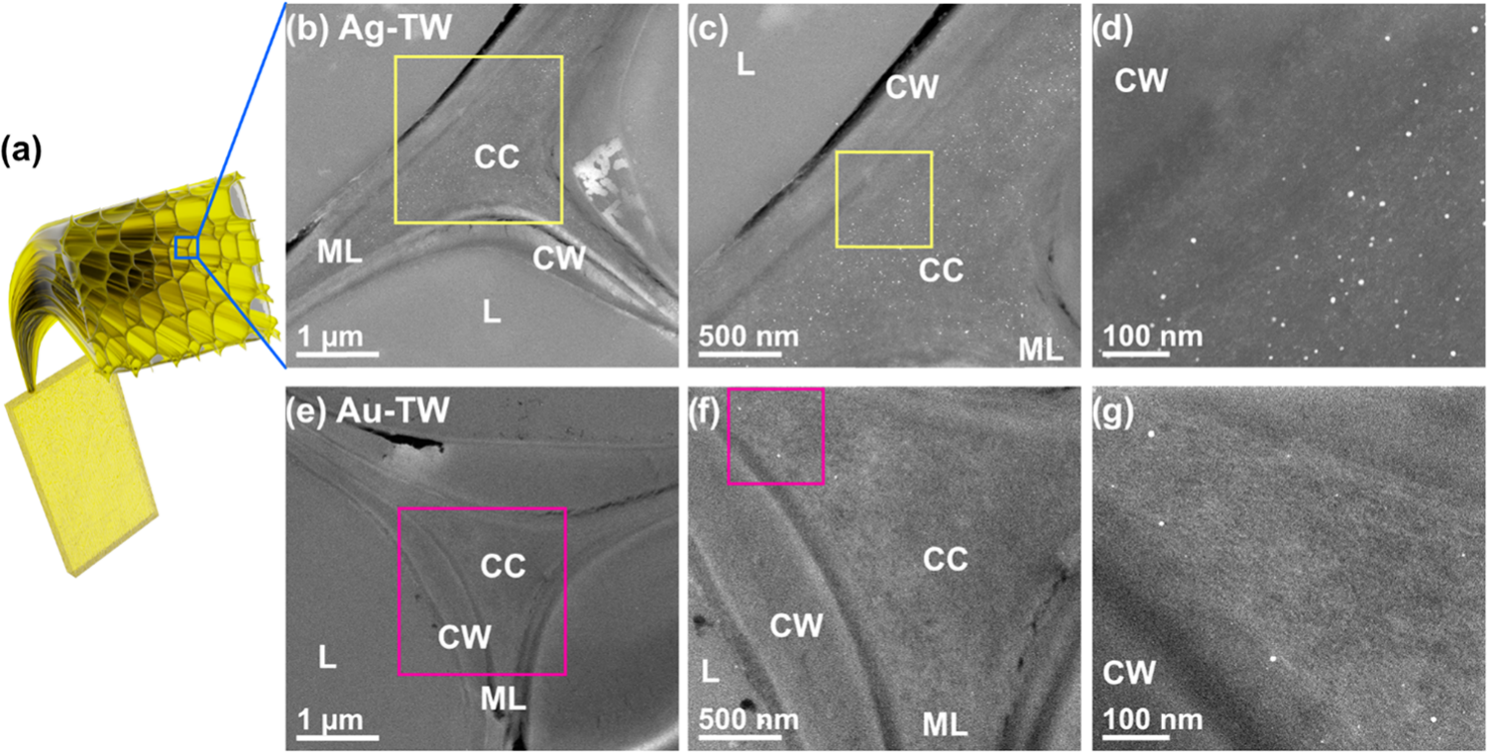
(a) Illustration of wood structure, the green square highlights the area of interest. Cross-sectional ADF-STEM micrographs of (b–d) Ag-TW and (e–g) Au-TW sections. The cell wall (CW), cell wall corner (CC), middle lamella (ML), and lumen (L) are marked. Colored squares indicate magnified areas. Lumen is the empty space at the center of a fibrous wood cell.

The light polarization split between light perpendicular and parallel to the fibers is more pronounced for Ag-TW compared with Au-TW (Figure S3). For clarity, it is more instructive to compare the ratio of the polarization split for different materials instead of an absolute intensity of the polarized light components. Figure 2b displays the ratio of the polarization split, i.e., the ratio between the transmitted light of perpendicular and parallel polarizations. The transparency of the composites is demonstrated in Figure 3c and the set-up for polarization measurements is shown in Figure 3d. First, we note that structurally colored TW clearly displays a stronger polarization split at the SPR band of the added nanoparticles, compared to the reference TW. The effect of nanoparticles seems akin to the effect of optical antennas created by surface plasmons.^77^ The “base” polarization split created by pure TW (green lines in Figure 2b) is enhanced by nanoparticles, as their distribution depends on the anisotropic structure of wood. In other words, the wood substrate provides a predesigned anisotropic nanoparticle distribution. Materials with homogenously distributed PNPs would not exhibit a polarization effect. The degree of enhancement depends on the specific nanoparticles, and again, is much more pronounced for Ag-TW than Au-TW. The silver nanoparticles make a significant contribution to the observed effect, whereas gold only slightly prevails over the neat reference TW (Figure 2b). This is linked to the higher particle content in Ag-TW and higher electrical conductivity of silver.^34^

### Composite Structure

The structure of Ag-TW and Au-TW was investigated using ADF-STEM imaging of sample cross-sections. Micrographs are presented in Figure 3. The cell wall corners are lignin-rich and degraded during delignification,^8^ but the hierarchical structure of interconnected wood fibers is still retained as the middle lamella is only partially degraded. The lumen (empty space in wood cells), cell wall corners, and voids in the middle lamella were fully impregnated with the polymer matrix. Visible gaps between the solidified polymer matrix and cell walls are artifacts from ultra-microtomy sectioning.

The nanoparticle distribution and separation at the nanoscale are of key importance for plasmonic coloration, since SPRs are sensitive to particle aggregation.^32^ Metal nanoparticles are highly contrasted against organic compounds in ADF-STEM imaging (Figure 3b–g). Spherical nanoparticles of silver and gold, in the size range of 3–12 nm, are observed in the TW biocomposite structures. Silver nanoparticles are primarily found between wood cells: in the cell wall corner and in the middle lamella between fiber cells. The particles are visibly separated and well-dispersed within these regions, and little to no coloration appears to be lost from particle aggregation. The particle distribution follows the structure of wood and forms tubular shells on the “fiber tubes”, effectively producing a “wood structure” of light-interacting particles. Optical effects related to the structure are, thus, enhanced by the incorporation of PNPs. The strong ratio of polarization split exhibited by Ag-TW (Figure 2b) is therefore attributed to the particle distribution, which was predesigned by the structure of the wood substrate.

Gold nanoparticles were present at a lower concentration, and also exhibited preferential distribution in the cell wall corners and the middle lamella, but with fewer particles. Nanoparticle aggregation is avoided, since the particles are visibly separated. A few gold nanoparticles were found inside the secondary wood cell wall, and additional nanostructural details are observable in Figure S4.

### Substrate Structure

**Figure 4:**
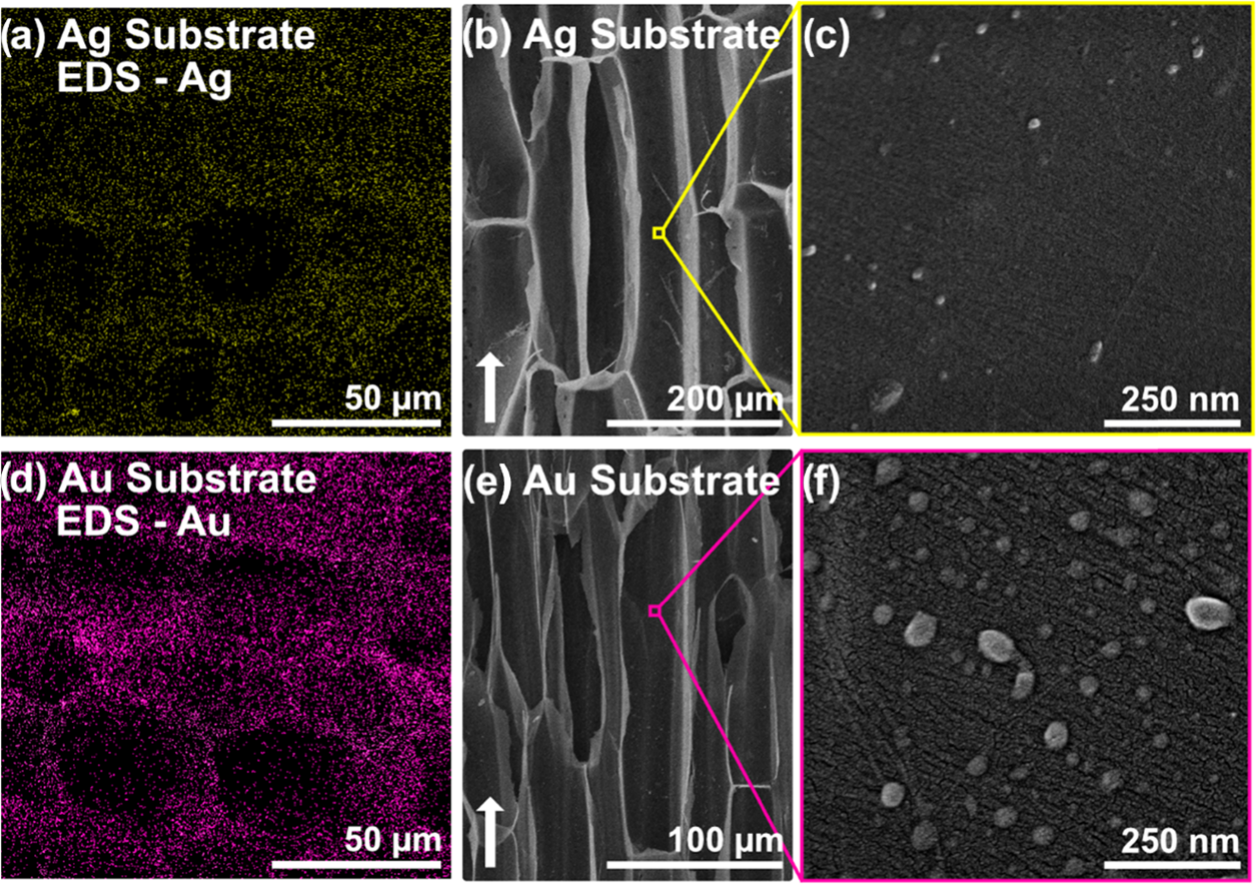
Cross-sectional EDS maps of (a) silver and (d) gold in respective substrates. SEM micrographs of the radial surface (white arrows indicate the fiber direction) of (b, c) silver and (e, f) gold-containing substrates, with higher magnifications show the inside of the fiber cell wall facing the “empty” lumen space.

The particle distribution and composition of substrates containing nanoparticles were investigated by EDS mapping of cross-sectional fracture surfaces. Mapping of gold and silver, in their respective substrates after microwave-assisted synthesis (Figure 4a,d), confirms a comparably homogeneous distribution of particles in the substrates on the scale of 100 μm. The respective EDS spectra are available in the Supporting Information (Figures S5 and S6). The process of mobile metal ion infiltration followed by in situ reduction facilitates homogeneous particle distribution. If pre-made, liquid-dispersed nanoparticles are used to impregnate wood substrates, uneven or incomplete infiltration may become a problem simply because of their larger size compared with ions. In Figure 4, the wood structure is clearly visible from the EDS signals, since particles are deposited on the cell wall, demonstrating the predesigning function of the wood substrate.

The elemental compositions of the substrates are presented in Table 2. The substrates attained nanoparticle contents of 2.1 wt % silver and gold, separately. This value matches the reported content in a silver-decorated wood filter.^46^ Assuming similar densities between nanoparticles and bulk materials, the respective substrates contain 0.30 vol % silver and 0.16 vol % gold, resulting in 0.02 vol % silver and 0.01 vol % gold in the final composites, Ag-TW and Au-TW. The lower SPR extinction and lower ratio of polarization split of Au-TW (Figure 2b) in comparison with Ag-TW, stem from the lower particle content, coupled with the lower electrical conductivity of gold.^34^ The chlorine content in both substrates is mainly caused by the delignification process.

**Table 2:** Elemental Composition of Substrates Containing Nanoparticles^a^

element	gold substrate [%]	silver substrate [%]
gold/silver	2.1	2.1
carbon	69.8	66.4
oxygen	25.9	28.2
chlorine	2.0	3.0

a Data presented in weight percentages, measured with EDS mapping.

Nanoparticle and substrate structures were investigated by SEM-imaging of cut surfaces. Micrographs of radial surfaces are presented in Figure 4. The hierarchical wood structure, on micro- and nanoscale, was retained for the substrates after in situ synthesis of PNPs.

The microstructure of interconnected fibers is visible at lower magnification. Higher magnification micrographs of the cell wall facing the empty lumen in wood revealed fibrils with retained structure and orientation.

Nanoparticles were observed on the lumen facing the cell wall of respective substrates (Figure 4c,f) and were larger in comparison with particles observed in the middle lamella and cell wall corners of TW composites (Figure 3). The difference may be caused by the larger void cavity of the lumen, resulting in a larger reservoir of the metal salt and continued growth during nanoparticle synthesis. Larger particles are fewer and have lower probability to be located in thin sections used for ADF-STEM imaging. The strategy to precipitate PNPs inside the wood structure is successful, and infiltration-related challenges of preformed PNPs are avoided.^78^

### Reduction Mechanism for Nanoparticle Synthesis

**Figure 5:**
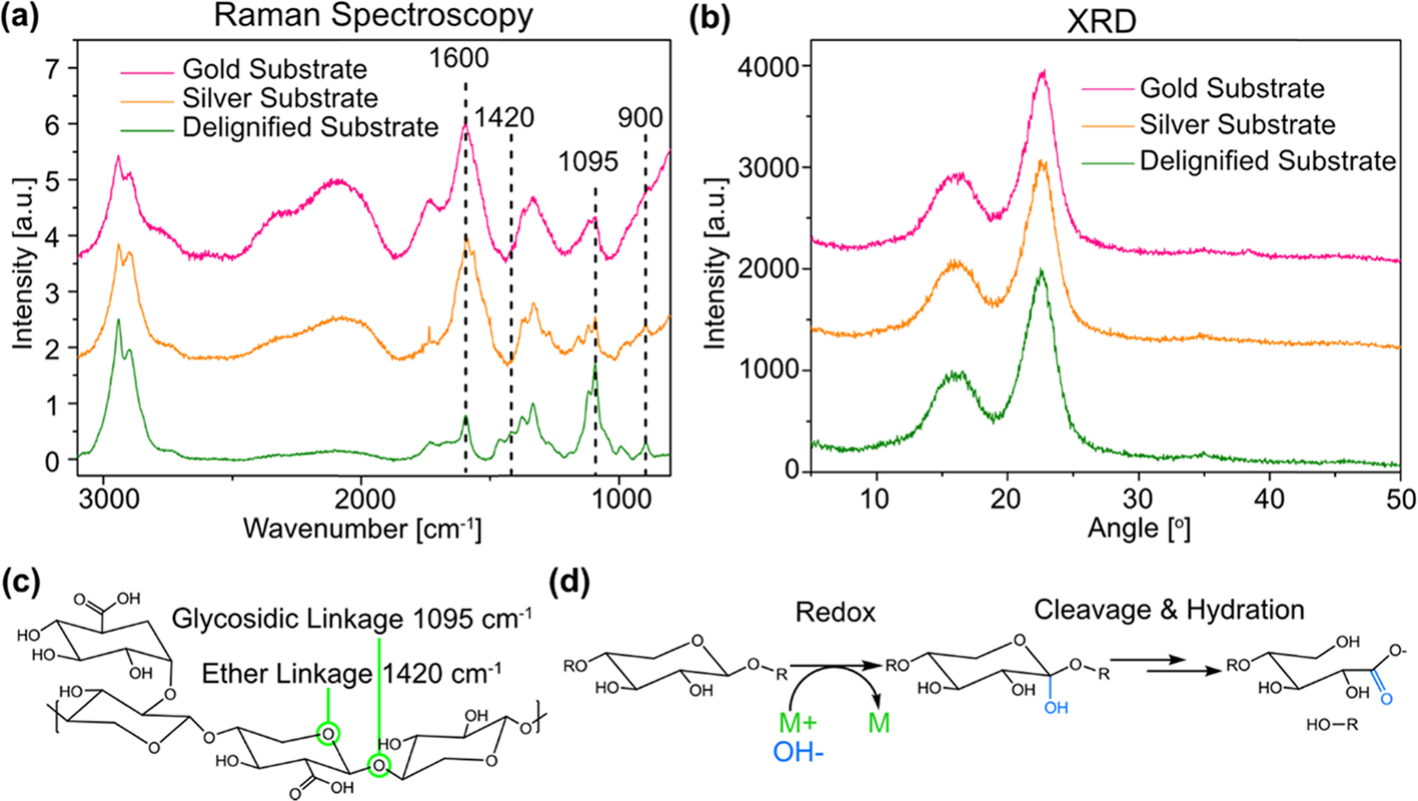
(a) Raman (normalized to CH_2_ vibrations at 1333 cm^–1^) and (b) XRD spectra of substrates before and after nanoparticle synthesis. (c) Schematic of xylan (common hemicellulose) with highlights of the glycosidic and ether linkages with their respective Raman active wavenumber. (d) Schematic of the proposed mechanism: redox reaction involving oxidizing the polysaccharide C1 carbon and reducing metal ions, followed by cleavage of the glycosidic linkage and hydration to a carboxylate group.

The wood tissue serves as a reducing agent, which makes the present process environmentally benign compared with those using common reduction agents, e.g., sodium boron hydride. The mechanism for the reduction of metal salts to PNPs in delignified wood substrates was therefore investigated with vibrational spectroscopy. Superficial confocal Raman measurements could detect differences in the vibrational spectra, which were not observed in FTIR bulk measurements (Figure S7). The reason is that the reactions were primarily active on cell wall surfaces. The Raman spectra (normalized to CH_2_ vibrations at 1333 cm^–1^) of substrates before and after nanoparticle synthesis are presented in Figure 5a.

The reduction of both metal nanoparticles follows similar oxidative paths: glycosidic (1095 cm^–1^) and ether linkages (1420 cm^–1^) of the substrate are cleaved as carboxylate groups form (1600 cm^–1^).^79−81^ The generation of carboxylate groups is observed as the growth and broadening of the overlapping peak of aryl stretching (1602 cm^–1^)^80^ from residual lignin in the wood template. The width of the carboxylate peak is related to various structures coordinating with metal nanoparticles.^81^ The coordinating structures attract the nanoparticles to the cell wall, so that particle aggregation is limited. The strong particle connection to the substrate makes it possible to wash, carry out solvent exchange, and infiltrate polymer precursors without loss of nanoparticles. Schematics of common hemicellulose (xylan) and the proposed mechanism is shown in Figure 5c,d.

Glycosidic and ether linkages are found both in cellulose and in hemicelluloses, which are the amorphous polysaccharide compounds surrounding the nanoscale cellulose fibrils.^82^ The crystallinity indices (CI) of cellulose in substrates before and after nanoparticle synthesis were calculated from XRD measurements (Figure 5b), using the peak height method.^72^ Cellulose was largely unaffected by nanoparticle synthesis as the CI remained the same after the reactions, at 0.73. The oxidation, therefore, occurs primarily on superficial hemicelluloses, and possibly on amorphous cellulose. Some degradation of cellulose was observed for gold since the Raman active peak related to cellulose crystallinity decreased (900 cm^–1^,^79^Figure 5a). The same peak was unaffected for silver. This mechanism differs from studies where primary hydroxyls in cellulose nanocrystals^55^ or phenols in lignin^54,57^ are reducing metal salts.

## Conclusions

Plasmonic silver and gold nanoparticles were in situ synthesized in wood substrates by a low-temperature process to produce structurally colored transparent wood (TW), with load-bearing functions. The reinforcing wood substrate in TW brings additional functions during the processing of structurally colored TW. It serves as a green reducing agent and a predesigning scaffold, which ensures allocation of well-dispersed nanoparticles by substrate attachment. The nanoscale particle distribution was controlled by the substrate morphology, since the nanoparticles were formed on and inside the wood cell walls, effectively forming an anisotropic wood structure of nanoparticles. The light-interacting PNPs, are able to enhance structure-related optical properties, e.g., the polarization effect of TW. The thiol-ene polymer matrix not only provides optical transmittance and improves mechanical properties but also contributes to specific thiol-ene-related chemical stability of the PNPs. Our investigation demonstrates how structurally colored TW can be produced by facile means and shows the potential to fabricate anisotropic plasmonic nanocomposites based on wood, useful in load-bearing optical elements.

## Supporting Information Available

Photographs of templates after in situ synthesis of NPs; stress–strain curves from tensile tests; polarized transmittance of light; STEM micrograph of Au-TW; EDS spectra of each of silver- and gold-containing substrates (PDF)
