# Supplementary material for: Facile Processing of Transparent Wood Nanocomposites with Structural Color from Plasmonic Nanoparticles
Source: Chem Mater. 2021 May 4;33(10):3736–45. doi: 10.1021/acs.chemmater.1c00806 (PMC8158850; doi:10.1021/acs.chemmater.1c00806)
Supplement: Supplementary file 1 — cm1c00806_si_001.pdf [file cm1c00806_si_001.pdf]

Supporting Information

Facile processing of transparent wood nanocomposites with structural color from plasmonic nanoparticles

Martin Höglund[a], Jonas Garemark[a], Mathias Nero[b], Tom Willhammar[b], Sergei Popov[c], Lars A. Berglund*[,a]

[a]Department of Fibre and Polymer Technology, Wallenberg Wood Science Center, KTH Royal Institute of Technology, SE-100 44 Stockholm, Sweden.

[b]Department of Materials and Environmental Chemistry, Stockholm University, SE-106 91 Stockholm, Sweden.

[c]Department of Applied Physics, KTH Royal Institute of Technology, 114 19 Stockholm, Sweden.

*E-mail: blund@kth.se

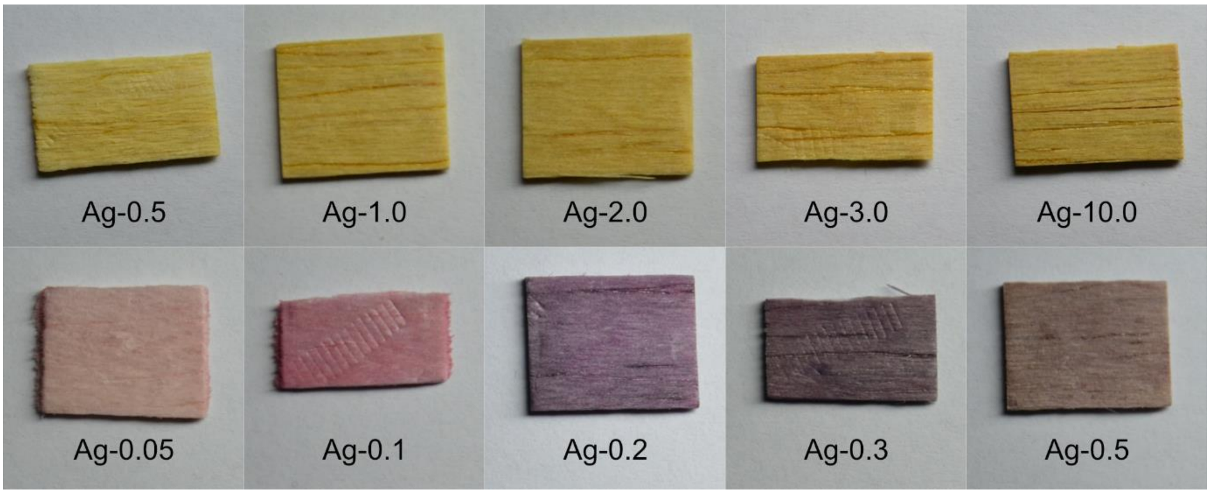

Figure S1. Photographs of templates after in-situ synthesis of NPs. Upper row, silver containing templates produced in solutions of 0.5 to 10.0 mM silver nitrate (left to right). Lower row, gold containing templates produced in solutions of 0.05 to mM chloroauric acid (left to right).

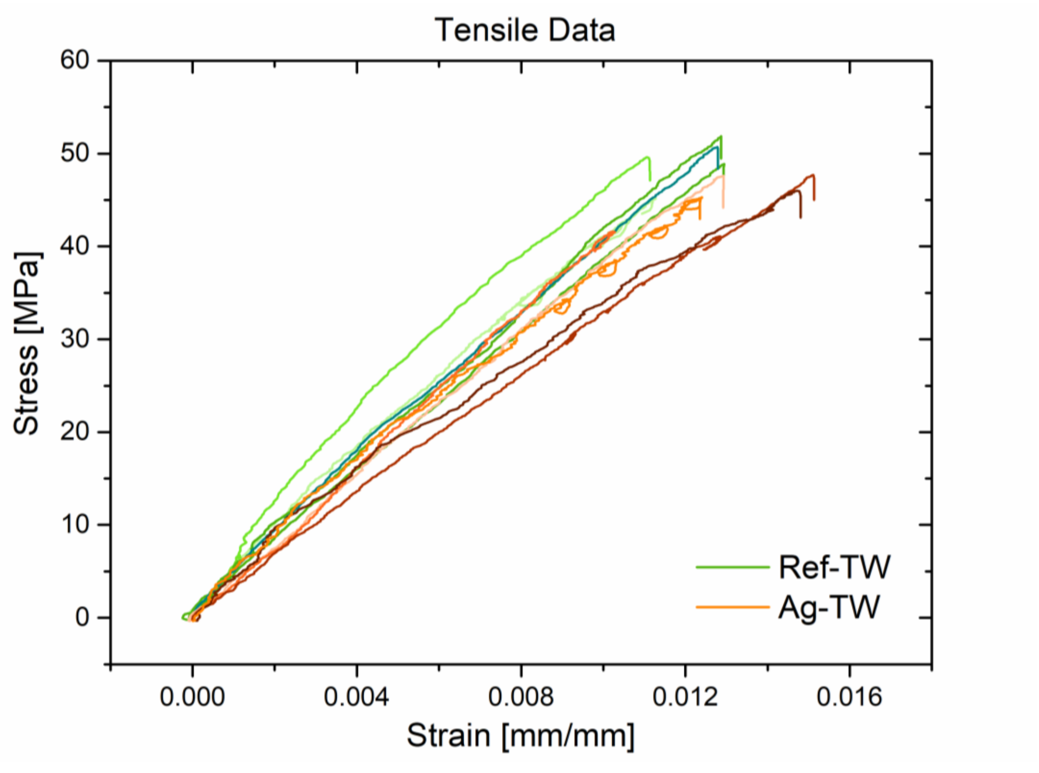

Figure S2. Stress-strain curves from tensile tests. Five samples were measured per composite.

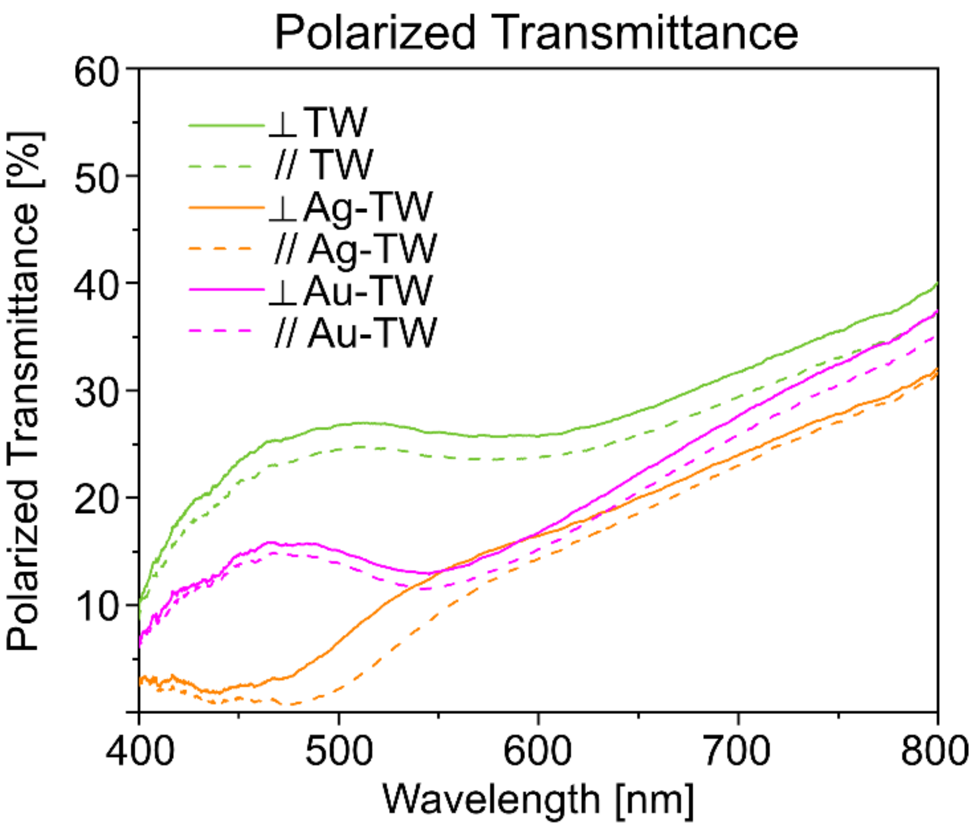

Figure S3. Polarized transmittance of light; perpendicular or parallel to the fiber direction of the wood substrate.

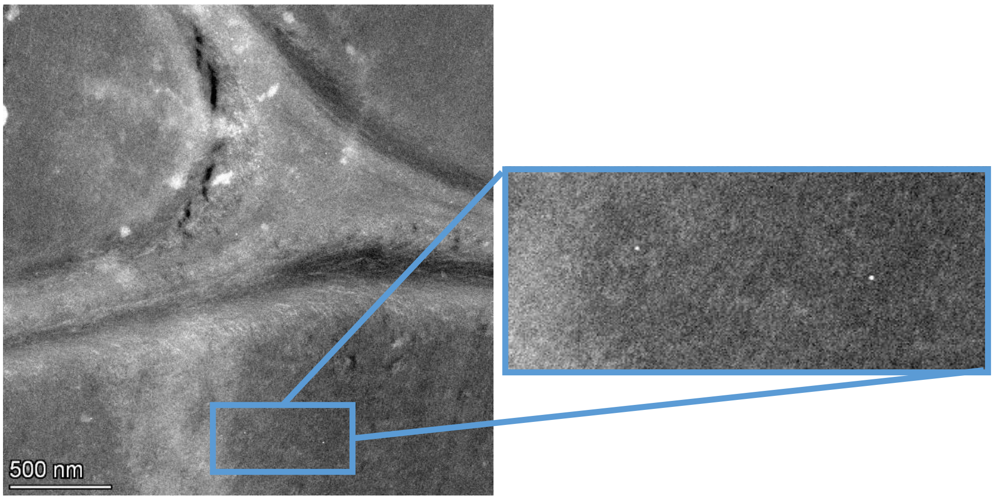

Figure S4. STEM micrograph of Au-TW revealing occasional nanoparticles inside the cell wall.

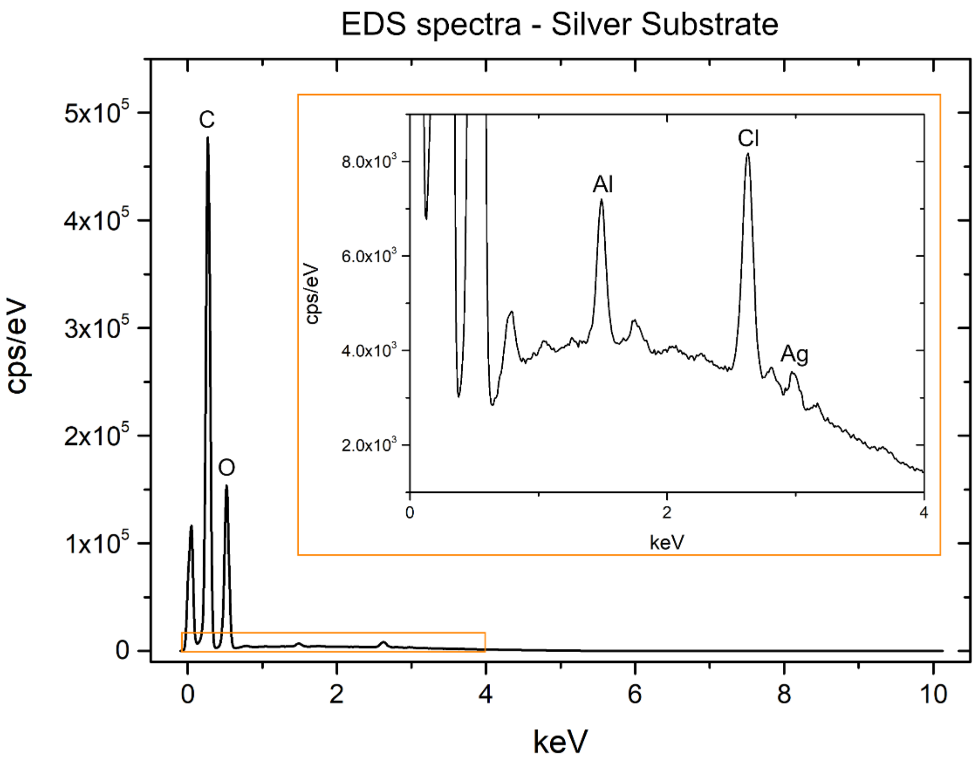

Figure S5. EDS mapping spectra of a silver substrate.

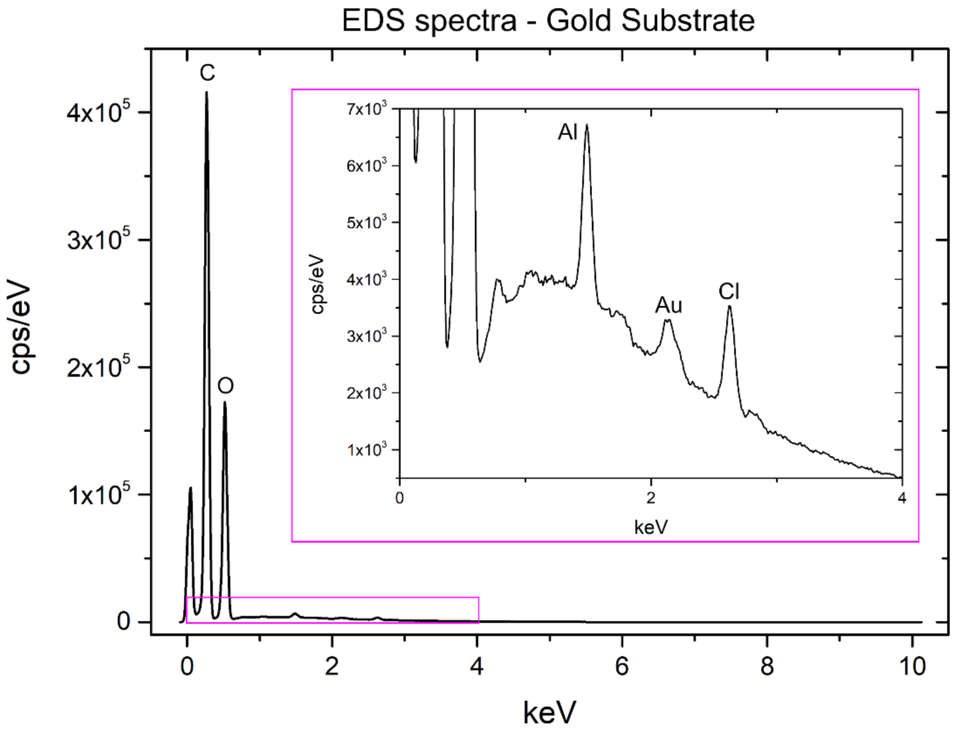

Figure S6. EDS mapping spectra of a gold substrate.

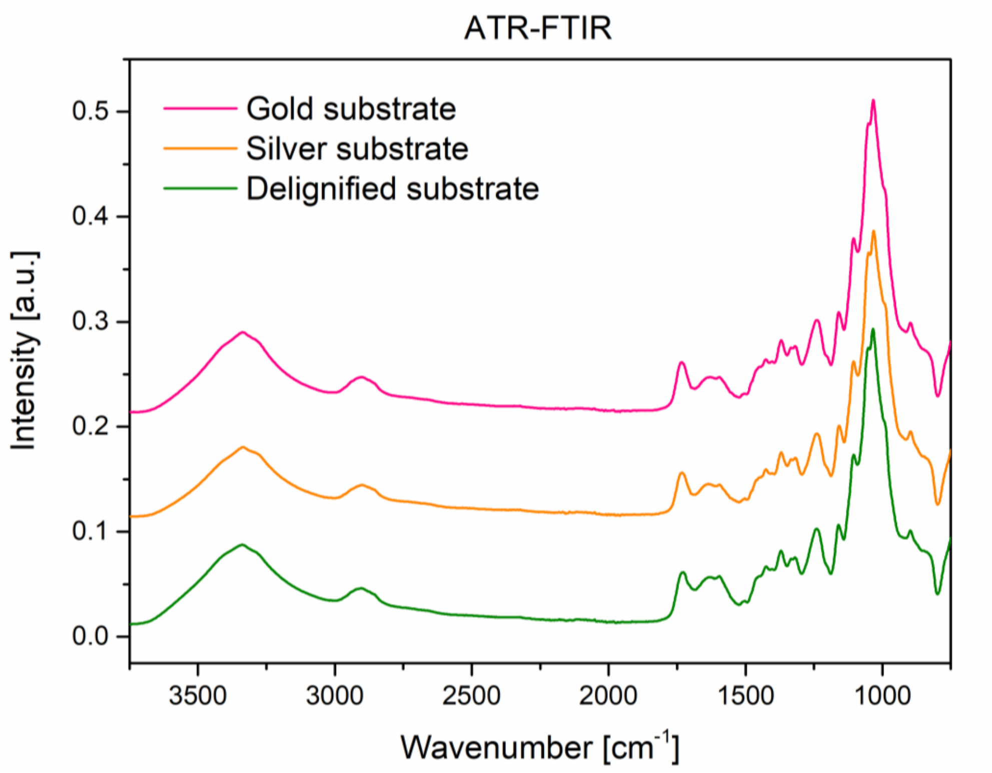

Figure S7. FTIR spectra of substrates. Before and after nanoparticle synthesis.
